# Antimicrobial resistance and genomic characterization of *Escherichia coli* from pigs and chickens in Zhejiang, China

**DOI:** 10.3389/fmicb.2022.1018682

**Published:** 2022-10-24

**Authors:** Wei Zhou, Rumeng Lin, Zhijin Zhou, Jiangang Ma, Hui Lin, Xue Zheng, Jingge Wang, Jing Wu, Yuzhi Dong, Han Jiang, Hua Yang, Zhangnv Yang, Biao Tang, Min Yue

**Affiliations:** ^1^Zhejiang Provincial Center for Animal Disease Prevention and Control, Hangzhou, China; ^2^State Key Laboratory for Managing Biotic and Chemical Threats to the Quality and Safety of Agro-Products, Institute of Agro-Product Safety and Nutrition, Zhejiang Academy of Agricultural Sciences, Hangzhou, China; ^3^School of Food Science and Technology, Jiangnan University, Wuxi, China; ^4^Key Laboratory of Marine Food Quality and Hazard Controlling Technology of Zhejiang Province, China Jiliang University, Hangzhou, China; ^5^The Institute of Environment, Resource, Soil and Fertilizers, Zhejiang Academy of Agricultural Sciences, Hangzhou, China; ^6^Zhejiang Provincial Center for Disease Control and Prevention, Hangzhou, China; ^7^Department of Veterinary Medicine, Institute of Preventive Veterinary Sciences, Zhejiang University College of Animal Sciences, Hangzhou, Zhejiang, China

**Keywords:** *Escherichia coli*, animal origin, antimicrobial resistance, genomic characterization, virulence genes

## Abstract

*Escherichia coli* is considered an opportunistic pathogen and an indicator for antimicrobial resistance (AMR) monitoring. Despite many reports on its AMR monitoring, studies based on genome-based analysis of AMR genes are still insufficient. Here, 181 *E. coli* strains were isolated from anal swab samples collected from pigs and chickens of animal farms located in Eastern China and sequenced through the Illumina platform. The results showed that 87.85% (159/181) of the *E. coli* isolates were multidrug-resistant (MDR). Ampicillin (AMP)- spectinomycin (SPT)- tetracycline (TET)- florfenicol (FFC)- sulfisoxazole (SF)- trimethoprim/sulfamethoxazole (SXT) was the predominant AMR pattern. By whole-genome sequencing, we found that ST10 (10.49%, 19/181) and ST48 (7.18%, 13/181) were major sequence types. IncFIB and IncX1 were the most prevalent plasmid replicons. The AMR genes *bla*_NDM-5_ (1.10%, 2/181), *mcr*-1 (1.10%, 2/181), *tet*(X4) (1.10%, 2/181), and *cfr* (6.08%, 11/181) were also found in these isolates. In addition, among the 169 virulence genes detected, we identified *ast*A (37.02%, 67/181), *hly*A (1.66%, 3/181), *hly*B (1.66%, 3/181) and *hly*D (1.66%, 3/181), which were closely related to heat-stable enterotoxin 1 and α-hemolysin. In addition, there were 33 virulence genes associated with the iron uptake system, and 46 were adhesion-related genes. Our study highlighted the need for routine surveillance of AMR with advanced genomic approaches, providing up-to-date data on the prevalence of AMR for the development and execution of antimicrobial stewardship policy.

## Introduction

The recent emergence and rapid increase of multi-drug resistant (MDR, resistance to more than three kinds of antibiotics) bacteria have caused public concern, represented by *Escherichia coli* resistant to carbapenem, colistin and tigecycline which were recognized as the last line of resort (Li et al., 2017; He et al., 2019; Ma et al., 2022). Due to incorrect use and misuse of antibiotics, the spread of antimicrobial resistance (AMR) is accelerating (Wang et al., 2022; Tang et al., 2022a,b). The continuous spread of AMR not only increases the difficulty of preventing and controlling livestock and poultry diseases but also seriously threatens livestock products’ safety and endangers consumers’ health (Xu et al., 2022; Li et al., 2022a).

*E. coli* is a commonly used AMR indicator in human and food animal (Brisola et al., 2019; Ma et al., 2022). There have been many reports on the study in *E. coli* and monitoring of the spread of AMR. The emergence of plasmid-mediated carbapenem resistance genes, especially *bla*_NDM_, has seriously affected the efficacy of meropenem (0.4%, 1/219; Tang et al., 2019, 2021b). Colistin resistance mediated by a plasmid-encoded *mcr-*1 was first documented in China during routine surveillance of food animals (21%, 166/804; Liu et al., 2016). A retrospective survey showed that *mcr*-1 was first traced back to 1980 but was not prevalent among bacteria until 2009 (Shen et al., 2016). With the detection rate of *mcr*-1 in bacteria increasing year by year, a relatively high occurrence rate of the *mcr*-1 gene (1%) was detected from *E. coli* in human (Wang et al., 2017). Tigecycline resistance are mediated by two novel genes, *tet*(X3) and *tet*(X4), both of which can significantly reduce the efficacy of tigecycline (He et al., 2019; Sun et al., 2019; Guan et al., 2022). Additionally, bacterial strains carrying the *cfr* gene encoding 23S rRNA methylase, which is resistant to five classes of antimicrobials, including phenols, lincosamides, oxazolidinones, pleuromutilin, and streptomycin A,allowing bacteria to develop MDR (Deng et al., 2014; Tang et al., 2022c).

Whole genome sequencing (WGS) played a vital role in AMR study (Tang et al., 2020b; Peng X. et al., 2022; Li et al., 2022b). We can obtain the tested strains’ AMR and virulence genes by combining them with the antimicrobial sensitivity test (AST). It is helpful to better understand AMR’s development and transmission (Teng et al., 2022). WGS has become an indispensable and reliable tool for revealing the AMR mechanism in global pathogen surveillance (Boolchandani et al., 2019; Tang et al., 2021a; Yang et al., 2022).

In this study, we investigated the prevalence of AMR *E. coli* in pigs and chickens from animal farms in Eastern China and evaluated the AMR phenotypes, genotypes, virulence genes, and plasmids replicons. This study helps understand the AMR situation and provides a reference to formulate livestock AMR control policies to better protect food safety in China.

## Materials and methods

### Sample collection and strain isolation

**Figure 1:**
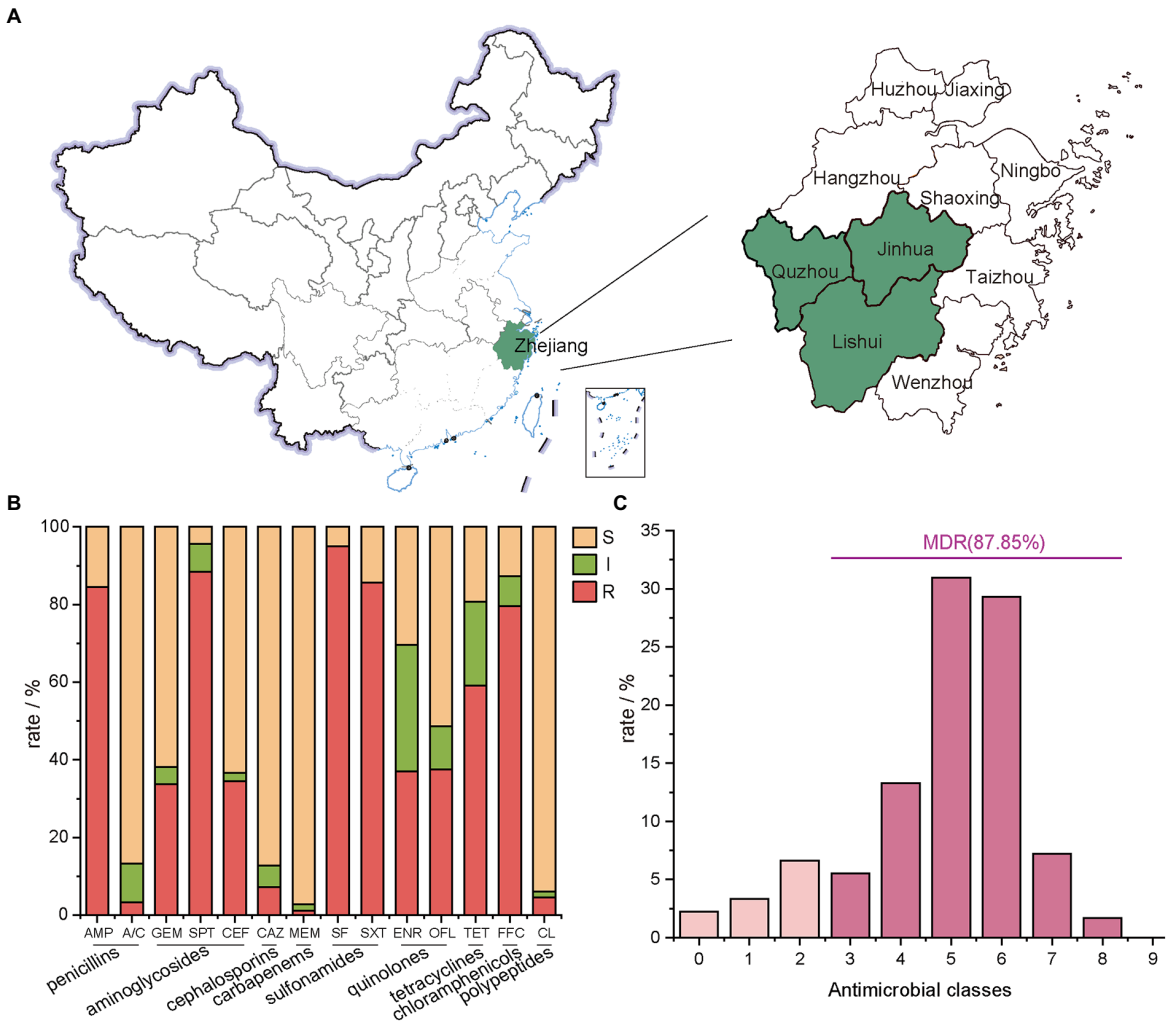
Geographical distribution of the sampling areas in Zhejiang Provinces, China, and AMR rates of *E. coli* isolates. **(A)** Sample sources of 181 strains of *E. coli,* Zhejiang Province in this study are shaded in green; **(B)** the number of *E. coli* isolates from farms in Zhejiang Province resistant to different antibiotics. In this experiment, 14 antibiotics with inflection points were divided into 9 categories; **(C)** the distribution of MDR strains.

A total of 200 anal swab samples were collected from three cities (Lishui, Jinhua, and Quzhou) located in Zhejiang Province, Eastern China, from March to April 2021. The samples were randomly collected from 110 chickens and 90 pigs in 5 poultry farms and 4 swine farms, respectively (Table 1; Figure 1A). All experiment activities in this study were approved by the Institutional Review Board of Zhejiang Academy of Agricultural Sciences.

**Table 1:** Source of 200 samples and isolation of 181 *E. coli* strains.

City	Number of farm	Animal	Number	Isolated strains	Separation rate (%)
Lishui	2	Chicken	50	46	92.00
	1	Pig	25	21	84.00
Quzhou	1	Chicken	20	19	95.00
	2	Pig	40	39	97.50
Jinhua	2	Chicken	40	31	77.50
	1	Pig	25	25	100.00

Anal swab samples were enriched in 10 ml of buffered peptone solution (BPW, Land Bridge, Beijing, China). After initial pre-enrichment in BPW, 0.1 ml of enriched samples were streaked on MacConkey Agar (Land Bridge, Beijing, China) or Eosin Methylene Blue Agar (Land Bridge, Beijing, China) and incubated at 37°C for 24 h. Only one suspicious colony (round, moist, and show pink color on MacConkey Agar; purple-black color with green metallic sheen on EMB Agar) were selected and further cultured on Luria-Bertani (LB) Agar (Land Bridge, Beijing, China). Bacteria identification was carried out by MALDI-TOF MS. Confirmed isolates were stored at −80°C.

### Antimicrobial sensitivity testing

Micro-broth dilution method was used (Bio Fosun, Fosun Diagnostics, Shanghai, China) to determine the AMR profile of *E. coli* isolates (Tang et al., 2022b). The panel of antimicrobial compounds tested included ampicillin (AMP), augmentin (amoxicillin/clavulanic acid, A/C), gentamicin (GEM), tetracycline (TET), spectinomycin (SPT), florfenicol (FFC), sulfisoxazole (SF), trimethoprim/sulfamethoxazole (SXT), ceftiofur (CEF), ceftazidime (CAZ), enrofloxacin (ENR), ofloxacin (OFL), meropenem (MEM), ampicillin (APR), colistin (CL) and mequindox (MEQ). The 14 tested antibiotics are grouped into 9 classes (Figure 1B), including penicillins (AMP and A/C), aminoglycosides (GEM、SPT, and CEF), cephalosporins (CAZ), carbapenems (MEM), sulfonamides (SF and SXT), quinolones (ENR and OFL), tetracyclines (TET), chloramphenicols (FFC) and polypeptides (CL). The breakpoint for each antimicrobial was from the Clinical and Laboratory Standards Institute (CLSI, 2016:M100-S30). *E. coli* ATCC 25922 was used as quality control.

### Whole genome sequencing and bioinformatics analysis

The genomic DNA extraction of *E. coli* was performed using a bacterial DNA extraction kit (Generay, Shanghai, China). The whole genome sequencing was performed on the Novaseq 6,000 (Illumina, SanDiego, CA, United States). Clean reads were assembled using SPAdesv3.12.0 (Bankevich et al., 2012). Genome annotation was performed using the NCBI Prokaryotic Genome Annotation Pipeline (Tatusova et al., 2016). ABRicate 1.0.1 tool^1^ and VFDB database were applied to predict the virulence genes and AMR genes. Replicons and sequence type (ST) were determined at the Center for Genomic Epidemiology (CGE).^2^ Phylogenetic analysis of the genome and plasmids was performed by kSNP 3.1 software based on the maximum-likelihood method (Gardner et al., 2015). Easyfig 2.2.5 was used for comparative analysis of the plasmids (Sullivan et al., 2011).

### Statistical analysis

TBtools was used for clustering heat map analysis of AMR genes, AMR phenotypes, virulence genes, and plasmid replicons (Chen et al., 2020).

## Results

### Prevalence and AMR of *Escherichia coli* isolates

A total of 181 *E. coli* strains were isolated from all 200 anal swab samples with a detection rate of 90.50%. Among them, 96 isolates were from chickens, and 85 isolates were from pigs (Table 1).

**Figure 2:**
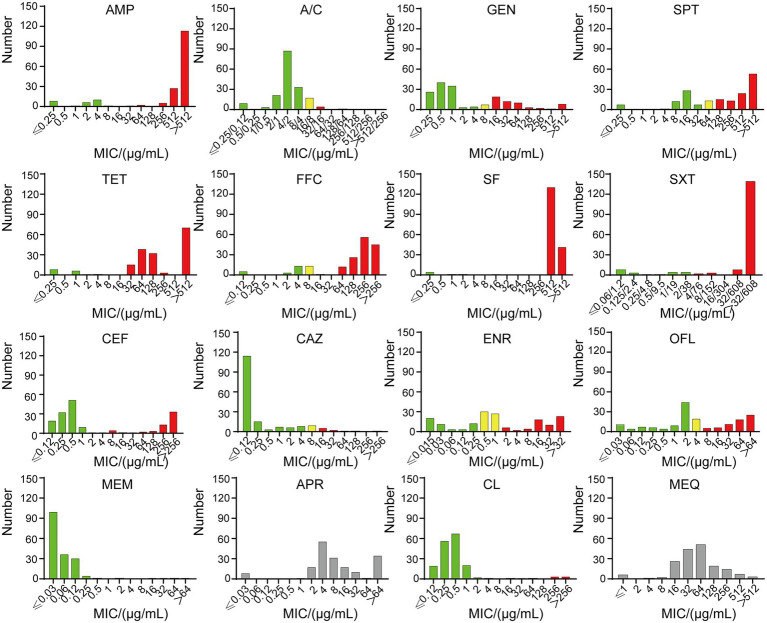
The MICs of 181 strains. Green indicates sensitivity, yellow indicates intermediary, and red indicates resistance. SF and SXT have no intermediate value, and APR and MEQ have no breakpoint. Ampicillin (AMP), amoxicillin/clavulanic acid (A/C), gentamicin (GEM), tetracycline (TET), spectinomycin (SPT), florfenicol (FFC), sulfisoxazole (SF), trimethoprim/sulfamethoxazole (SXT), ceftiofur (CEF), ceftazidime (CAZ), enrofloxacin (ENR), ofloxacin (OFL), meropenem (MEM), apramycin (APR), colistin (CL) and mequindox (MEQ).

*E. coli* isolates showed the lowest resistance rate to MEM at 2.76%, followed by A/C at 3.31%, CL at 5.52%, and CAZ at 7.18% (Figure 1B). A high resistance rate was shown for five antibiotics in descending order. They were SF (95.03%), SPT (88.40%), SXT (85.64%), AMP (84.53%) and FFC (79.56%). Except for TET with a resistance rate of 59.12%, the resistance rates of *E. coli* isolates to OFL, ENR, CEF and GEM ranged between 35.0% ~ 40.0% (Figure 2).

The 14 tested antibiotics are grouped into 9 classes (Figure 1B), including penicillins (AMP and A/C), aminoglycosides (GEM、SPT, and CEF), cephalosporins (CAZ), carbapenems (MEM), sulfonamides (SF and SXT), quinolones (ENR and OFL), tetracyclines (TET), chloramphenicols (FFC) and polypeptides (CL). Carbapenems class of antibiotics, which in this study is MEM had the lowest resistance rate. Meanwhile, the sulfonamides class of antibiotics had the highest resistance rate with 181 resistant isolates, of which 172 were resistant to SF.

87.85% of isolates were MDR (Figure 1C; Supplementary Table S1), and the predominant MDR pattern (19.50%, 31/159) was resistance to AMP-SPT-TET-FFC-SF-SXT. Notably, three strains were determined to be resistant to 12 types of antibiotics. The patterns were AMP-A/C-GEM-SPT-TET-FFC-SF-SXT-CEF-CAZ-ENR-OFL and AMP-GEM-SPT-TET-FFC-SF-SXT-CEF-CAZ-ENR-OFL-CL, respectively.

### Genomic characterization of *Escherichia coli* isolates

**Figure 3:**
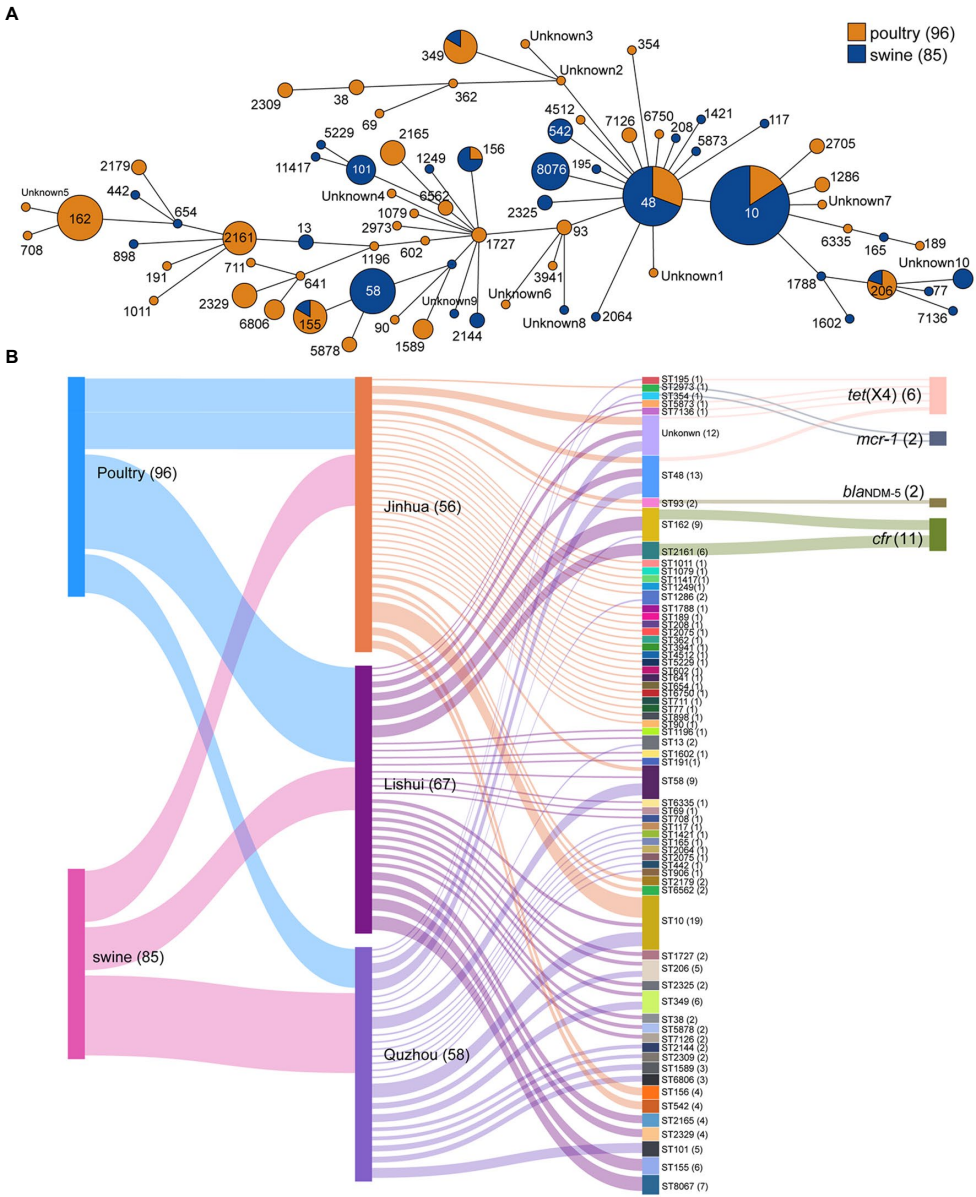
Minimum spanning tree of *E. coli* strains based on MLST and Sankey diagram combining the provinces, STs, farms, and sampling sources based on 181 *E. coli* isolates. **(A)** Each node represents a single ST. The size of the nodes is proportional to the number of isolates. The length of branches between each node is proportional to the number of different alleles that differ between two linked nodes; **(B)** the diameter of the line is directly proportional to the number of isolates, which is also marked with numbers. The lines are colored according to the city and the sampling source.

Sixty-five different sequence types (STs) were generated in 181 *E. coli* isolates (Figure 3), which were further grouped into 16 clonal complexes (CCs) and 38 singletons. Among them, ST10 was most prevalent with 19 isolates (10.50%), followed by ST48 with 13 isolates (7.18%), ST58 and ST162 both with 9 isolates (4.97%).

**Figure 4:**
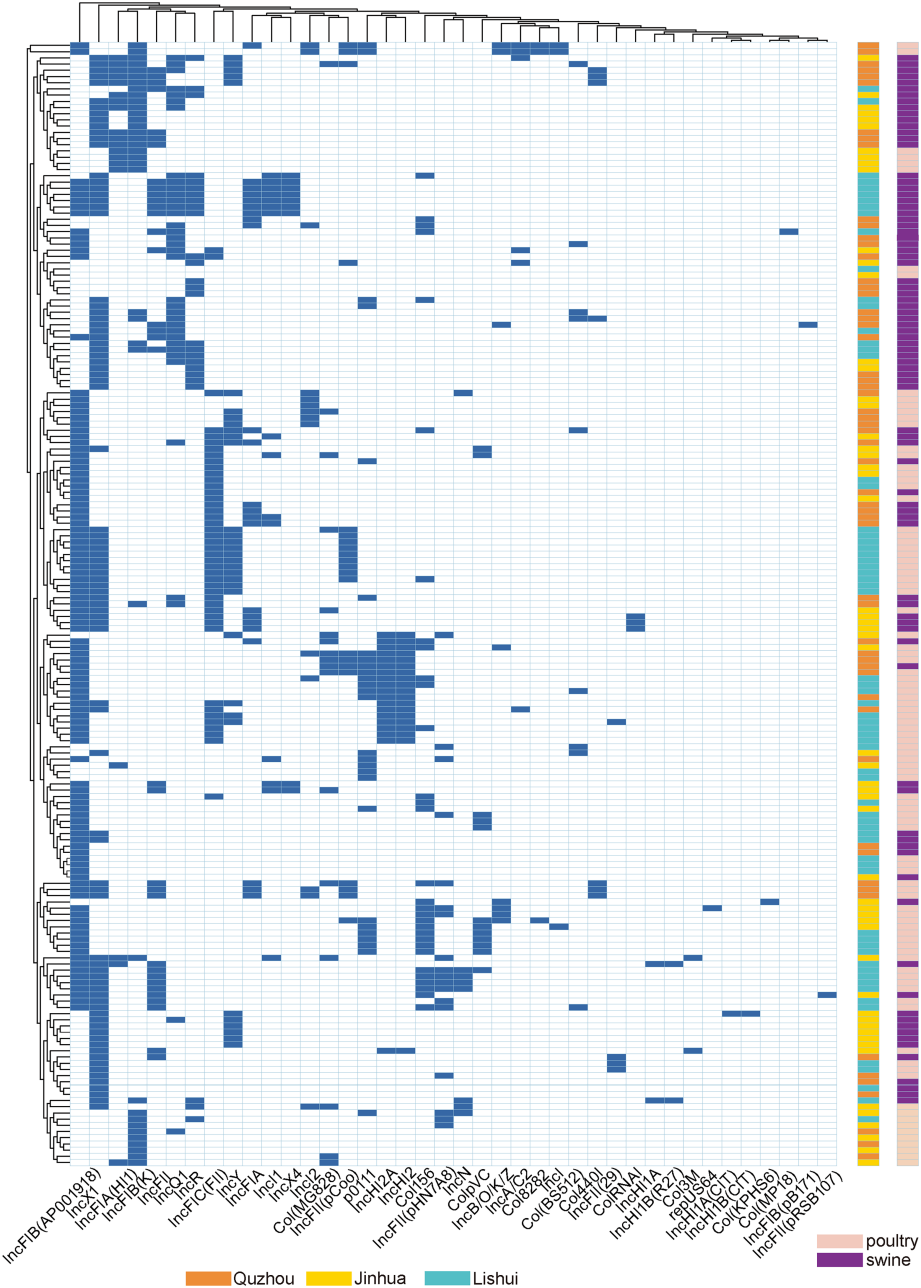
Dendrogram of hierarchical clustering heatmap of the 181 isolates and 40 plasmid replicons. Y-axis is the isolate number, and x-axis is the selected resistance genes identified. Non-expression of resistance is a white background and resistance phenotype is a blue background. Orange indicates isolates were isolated from Quzhou samples, aqua blue indicates isolates were isolated from Lishui, and bright yellow indicates isolates were isolated from Jinhua. Purple indicates that the strain is isolated from pig anal swab samples, and green indicates that the strain is isolated from bird anal swab samples.

The plasmid replicon analysis (Figure 4) showed that a total of 40 types of plasmid replicons were detected in all 181 *E. coli* isolates, of which 107 (59.12%) carried the IncFIB (AP001918) replicon, followed by IncX1 replicon existing in 86 isolates (47.51%). 94.48% (171/181) of the isolates carried 2 ~ 6 replicons. Similar plasmid replicon types can be found in different cities and animals, suggesting that plasmids carrying AMR genes may be widely spread through horizontal gene transfer.

**Figure 5:**
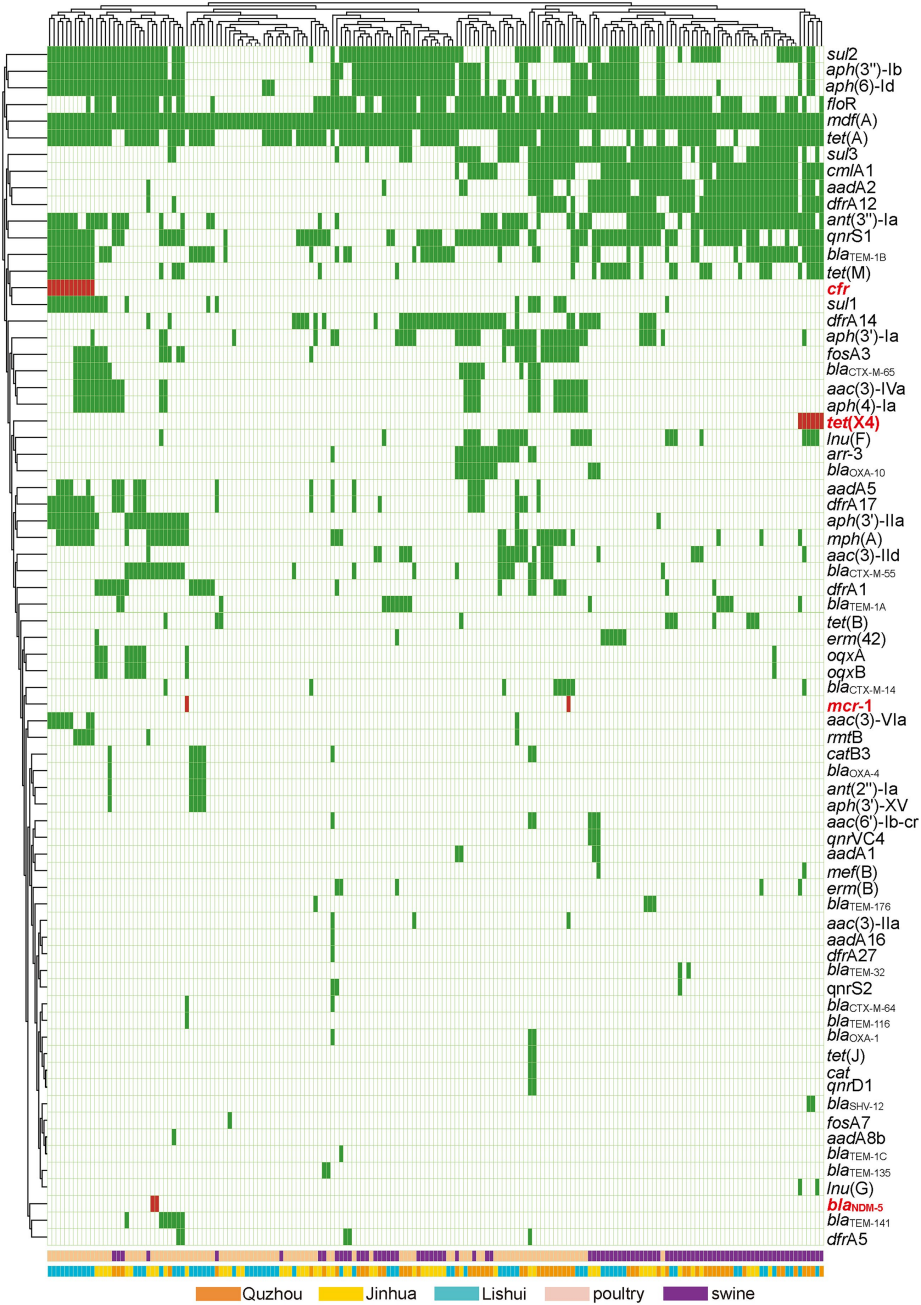
Antibiotic resistance patterns of *E. coli* isolates. Y-axis is the number of isolated strains, and x-axis is the selected AMR genes identified. Green indicates that the isolate is isolated from Quzhou sample, light blue indicates that it is isolated from Lishui, and light yellow indicates that it is isolated from Jinhua. Flesh color indicates that the isolate is isolated from poultry anal swab samples, and cinnabar indicates that it is isolated from pig anal swab samples. Dark green in the small grid indicates the presence of AMR genes, white indicates no, and red indicates the presence of risk AMR genes. The risk AMR genes mentioned here refer to the genes that have been focused on, such as *tet*(X4) (resistant to tigecycline), *mcr-1* (resistant to colistin), *bla*_NDM-5_ (meropenem) or *cfr* (resistant to oxazolidinone, amphenicol, lincosamide), which pose a serious threat to public health and safety. Rifamycin *(arr-3)*, Aminoglycoside (*aac(3)-Iia*, *aac(3)-Iid*, *aac(3)-Iva*, *aac(3)-Via*, *aac(6′)-Ib-c*r, *aadA16*, *aadA1*, *aadA2*, *aadA5*, *aadA8b*, *ant(2″)-Ia*, *ant(3″)-Ia*, *aph(3″)-Ib*, *aph(3′)-Iia*, *aph(3′)-Ia*, *aph(3′)-XV*, *aph(4)-Ia*, *aph(6)-Id*, *rmtB*), Beta-lactam (*bla*_CTX-M-14_, *bla*_CTX-M-55_, *bla*_CTX-M-64_, *bla*_CTX-M-65_, *bla*_NDM-5_, *bla*_OXA-10_, *bla*_OXA-1_, *bla*_OXA-4_, *bla*_SHV-12_, *bla*_TEM-116_, *bla*_TEM-135_, *bla*_TEM-141_, *bla*_TEM-176_, *bla*_TEM-1A_, *bla*_TEM-1B_, *bla*_TEM-1C_, *bla*_TEM-32_), Amphenicol (*catB3*, *cat*, *cmlA1*, *floR*, *cfr*), Folate pathway antagonist (*dfrA12*, *dfrA14*, *dfrA17*, *dfrA1*, *dfrA27*, *dfrA5*, *sul1*, *sul2*, *sul3*), Macrolide (*erm(42)*, *erm(B)*, *mdf(A)*, *mef(B)*, *mph(A)*), Fosfomycin (*fosA3*, *fosA7*), Lincosamide (*lnu(F)*, *lnu(G)*), Polypeptides (*mcr-1*), Quinolone (*oqxA*, *oqxB*, *qnrD1*, *qnrS1*, *qnrS2*, *qnrVC4*), Tetracycline (*tet*(A), *tet*(B), *tet*(J), *tet*(M), *tet*(X)).

As shown in Figure 5, 72 acquired AMR genes were detected in this study, among which *mdf*(A) was carried by all isolates. Notably, two isolates carried carbapenem resistance gene *bla*_NDM-5_, two isolates carried colistin resistance gene *mcr*-1, six isolates carried tigecycline resistance gene *tet*(X4), and eleven isolates carried linezolid resistance gene *cfr.*

**Figure 6:**
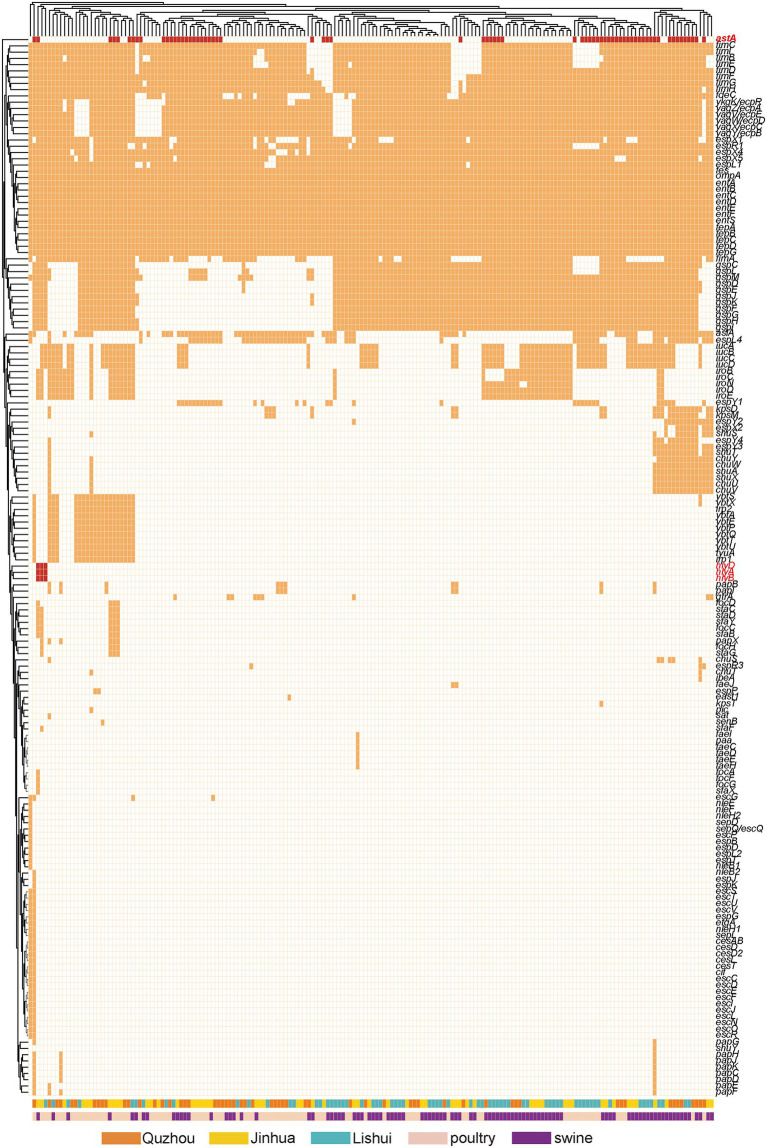
Dendrogram of the hierarchical clustering heat map of isolates and virulence genes. The figure shows the predicted virulence genes factor profile of the studied isolates. Y-axis is the isolate number and x-axis is the selected virulence genes identified. The red color in the small cells indicates virulence genes associated with toxin production, the orange color indicates genes editing other virulence factors, and the white color indicates no virulence expression. Withered grass color indicates isolates isolated from Quzhou samples, yellow indicates isolates from Jinhua, and blue color indicates isolates from Lishui. Incarnadine pink indicates that the isolates were isolated from pig anal swab samples, and bright green indicates that they were isolated from birds.

One hundred and sixty-nine virulence genes were found (Figure 6). Ten including *ent*A, *ent*B, *ent*D, *ent*E, *ent*F, *fep*A, *fep*C, *fep*D, *fes* and *omp*A were detected in all isolates. The virulence genes carried by the other isolates ranged from 25 to 77. The encoding gene *ast*A of heat-stable enterotoxin1 (East1) was detected in 37.02% (67/181) of the isolates. Three α-hemolysin encoding genes: *hly*A, *hly*B and *hly*D, were present simultaneously in isolates ECLSZ21-06, ECQZZ21-39, and ECJHZ21-15. Thirty-three virulence genes detected here were related to the iron uptake system, and 46 were adhesion-associated genes.

**Figure 7:**
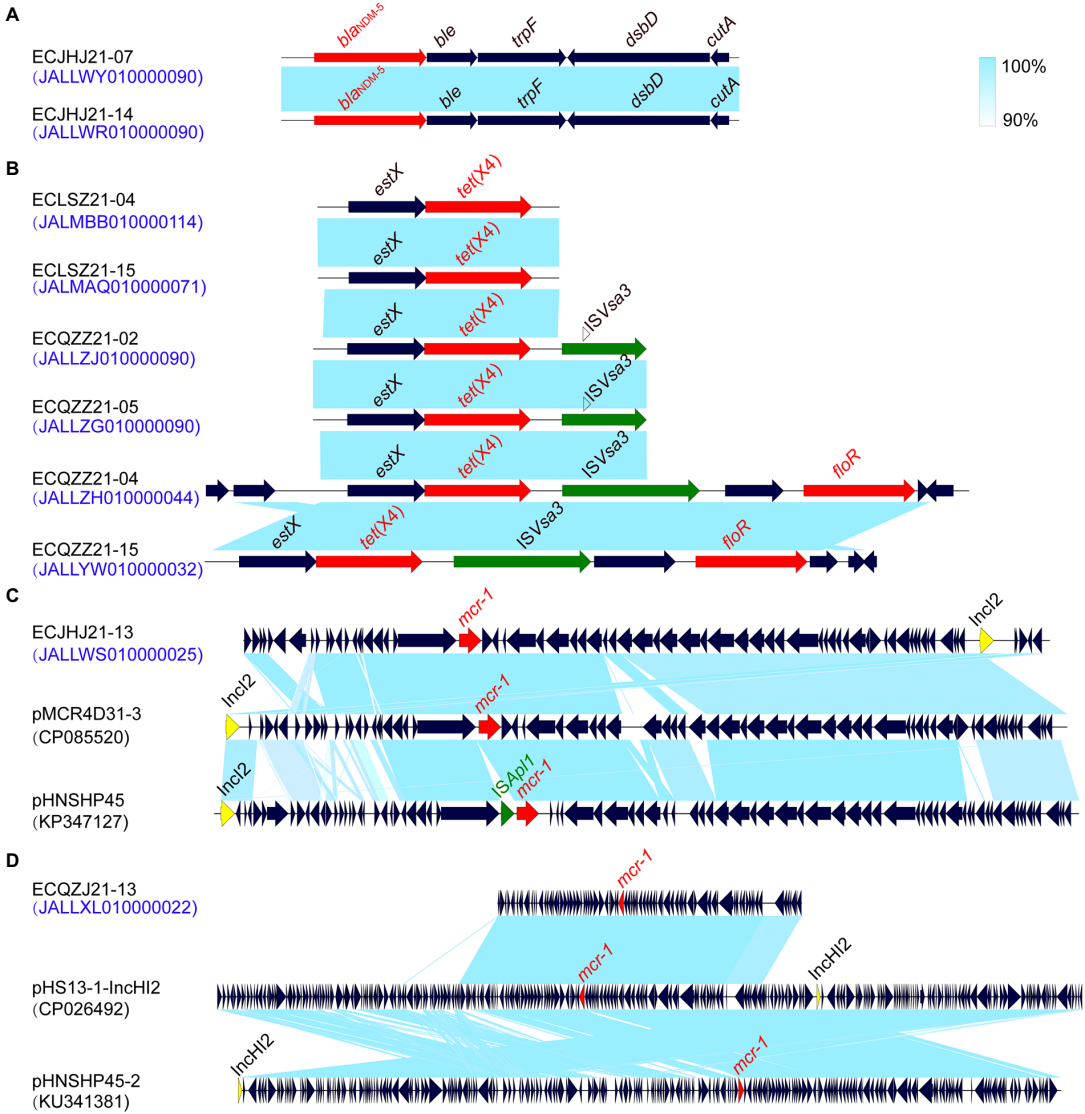
Comparison of the risk AMR gene environments in *E. coli* strains. **(A)** the *bla*_NDM-5_ gene environment. **(B)** the *tet*(X4) gene environment. **(C)** the *mcr-1* gene environment of IncI2 plasmids. **(D)** the *mcr-1* gene environment of IncHI2 plasmids.

In this study, we found that strains ECJHJ21-07 and ECJHJ21-14 carry the *bla*_NDM-5_ gene and have the same genetic context *bla*_NDM-5_-*ble*-*trpF*-*dsbD*. Strains ECLSZ21-04, ECLSZ21-15, ECQZZ21-02, ECQZZ21-05, ECQZZ21-04, and ECQZZ21-15 contain the *tet*(X4) gene, which is all adjacent to the *estX* gene. In the latter two strains, the *tet*(X4) gene is located in the gene arrangement *estX*-*tet*(X4)-IS*Vsa3* based on better sequencing quality. In addition, we found two strains carrying the *mcr-1* gene, which have different genetic environments. The *mcr-1*-carrying plasmid in strain ECJHJ21-13 is homologous with plasmids pMCR4D31–3 and pHNSHP45 with an IncI2 type; the fragment harboring *mcr-1* gene in strain ECQZJ21-13 is homologous with the IncHI2 plasmid (Figure 7).

**Figure 8:**
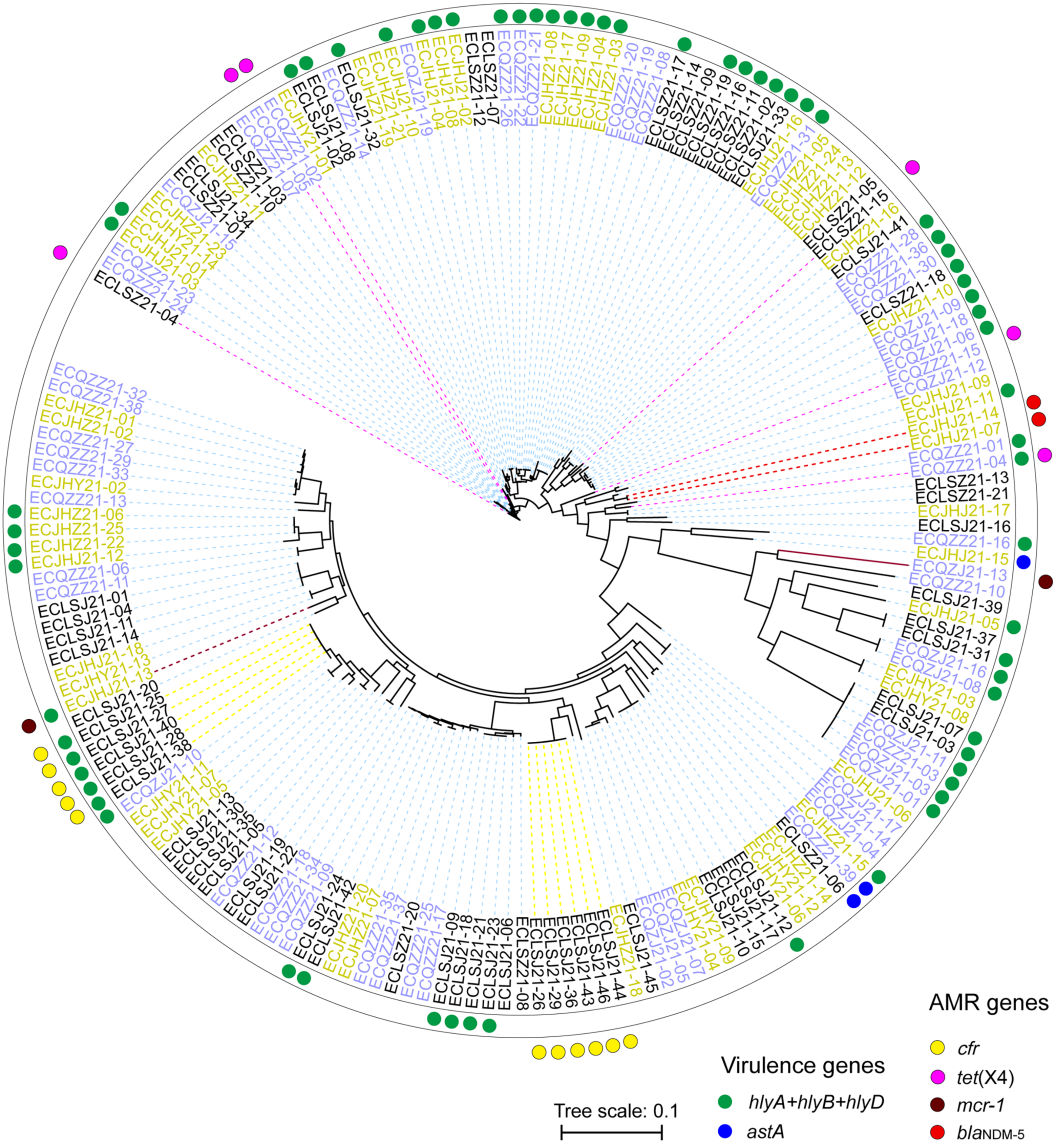
Phylogenetic SNP tree of *E. coli* strains with whole genome sequences. Red and blue dots indicate different virulence genes carrying strains, and yellow, pink, brick and red indicate different AMR genes carrying strains. The strains with different colors represent three different cities.

Strains from different cities can gather on the same branch. As the last line of drugs, the AMR genes *mcr-*1, *bla*_NDM_, and *tet*(X4) are distributed in different branches, showing different genetic relationships (Figure 8). In the present study, these AMR genes are more likely to be horizontal gene transfer between *E. coli* strains. In addition, the *cfr* gene mainly exists in two branches, which may be the clonal transmission (Figure 8).

### Analysis of genotypes and phenotypes of AMR *Escherichia coli* isolates

The highest concordance between genotype and phenotype was detected for carbapenems (100.00%), followed by polypeptides (93.92%), and tetracyclines (90.61%; Supplementary Table S2). In addition, two isolates carrying *bla*_NDM-5_ gene were found resistant to AMP, CEF, TET, FFC, SF, SXT, ENR, and OFL. All eleven isolates carrying the *cfr* gene showed resistance to AMP, SF, and SXT. Similarly, the isolates carrying *mcr*-1 showed resistance to AMP, TET, FFC, SF, CEF, ENR, and OFL, and the isolates carrying *tet*(X4) showed resistance to SPT, TET, FFC, SF, and SXT. These provide supporting evidence for AMR genes to explain drug resistance. The AMR gene *cfr* mainly existed in two STs, ST2161 and ST162. Two isolates carrying the *bla*_NDM-5_ were both assigned ST93. Isolates carrying *mcr*-1 belonged to ST2973 (ECJHJ21-13) and ST354 (ECQZJ21-13), respectively (Figure 3). Meanwhile, the isolates carrying *tet*(X4) demonstrated sequence type diversity, ST195, ST48, ST5873 and ST7136 were included.

## Discussion

The AMR of *E. coli* has become a worldwide public health problem (Rutuja et al., 2018). Pigs and poultry products may have a cross infection of AMR *E.coli* during processing and subsequent sale, which increases the risk of transmission and poses a significant threat to the sale of products and people’s health (Chang et al., 2020). To understand the current situation of AMR of *E. coli*, we analyzed AMR, MLST and virulence genes of *E. coli* isolated from anal swab samples of pigs and chickens in farms in Lishui, Quzhou and Jinhua, Zhejiang Province, Eastern China. In this study, relatively high AMR rates of FFC (79.56%), SXT (85.64%), SF (95.03%), SPT (88.40%) and AMP (84.53%) were detected that were consistent with the previously published results (Ma et al., 2022; Peng Z. et al., 2022). At the same time, the AMR rates of A/C (3.31%), MEM (2.76%), CL (5.52%) and CAZ (7.18%) were low. CL and MEM are recognized as the last line of defense against gram-negative bacteria (Lin et al., 2022; Wang et al., 2022) and low AMR rates reveal their resistance’s effectiveness is decreasing.

Genomic analysis revealed that the *E. coli* isolates harbored various AMR genes, which could be consistent with the AMR phenotype. All *E. coli* isolates contained the AMR gene *mdf*(A), and most of them also carried *tet*(A) (68.51%, 124/181), *flo*R (62.98%, 114/181) and *sul*2 (56.35%, 102/181). These genes mediate resistance to tetracycline, chloramphenicol, and sulfonamide antibiotics. These critical AMR genes in *E. coli* isolates from food animals present a tremendous public health concern. It’s important to mention that the acquired AMR genes in bacterial genomes do not inevitably confer phenotypic resistance and vice versa (Boolchandani et al., 2019; Tang et al., 2022a). Other mechanisms such as SNPs and MDRtransporter also significantly contribute to the phenotypic resistance (Boolchandani et al., 2019). The phenotypic confirmation is still essential for validating of AMR profiles. As we found in this study, only the acquired genes of carbapenem, colistin and tetracycline resistance have the highest consistency with the AMR phenotypes. While other acquired AMR genes can not predict the resistance phenotype of the bacteria very well.

Sixty-five different STs were determined in all *E. coli* isolates. Among them, ST10 accounted for the largest proportion, up to 10.50% (19/181). Next was ST48, and 13 isolates (7.18%) were tested for this ST type. ST10 *E. coli* has been seen repeatedly in related pathogenicity studies, with multiple disease outbreaks associated with ST10 in a broiler production unit by testing over 18 months (Bojesen et al., 2022).

Nine of the ST10 *E. coli* isolates detected in our study carried the virulence gene *ast*A, which plays an essential role in causing diarrhea and affecting the survival of animals (Meng et al., 2014; Paixão et al., 2016). In recent years, multiple researchers have found that some virulence genes can promote the AMR of bacteria strains (Fu et al., 2022). At the same time, some toxins will be enhanced under the pressure of antibiotics (Wangkheimayum et al., 2022). In our study, the prevalance of *ast*A is up to 36.81%. The *ast*A gene does not sufficiently lead to disease and is also found in healthy pigs, but when it cooperates with F pili or other virulence genes such as *sta* and *stb*, it will lead to diarrhea and pose a potential threat to food safety. Hemolysin is an important pathogenic factor of pathogenic *E. coli*. It not only plays a role in cracking cells but also promotes cell death by activating typical inflammatory bodies in cells. Hemolysin A (HlyA) toxin is important for triggering cell death in human macrophages(Verma et al., 2019; Gu et al., 2021). Our study obtained three isolates carrying encoding genes of three kinds of α-hemolysin, *hly*A, *hly*B, and *hly*D. Here, we would also like to note the proportion of virulence genes associated with the iron uptake system (33/169, 19.53%) and adhesion (46/169, 27.22%). Adhesins are usually a class of glycoprotein or glycolipid biomolecules that are mainly distributed on the surface of bacteria (Berne et al., 2015). The adhesins identified in this study included F1C hairs, K88 hairs, type I hairs, and *E. coli* common hairs.

*E. coli* isolates harbored several kinds of plasmids, with the dominance of IncFIB (AP001918) followed by IncX1. These plasmids have been verified linked to resistance to several antimicrobial classes, including *β*-lactams, aminoglycosides, sulfonamides, tetracyclines, etc. Our research also showed that these plasmids were carried by isolates originating from chickens and pigs in Zhejiang provinces, indicating that these plasmids could disseminate widely among different hosts (Hu et al., 2016; Tang et al., 2019, 2020a).

## Conclusion

In summary, a comprehensive study of AMR and genomic characterization of *E. coli* isolated from pigs and chickens anal swab samples in Eastern China was conducted. A high prevalence of MDR *E. coli* and many virulence determinants in the isolates posed a potential risk to food safety. This is a significant warning for public health safety in Zhejiang Province. It is essential to continue monitoring the MDR *E. coli* and implement antimicrobial stewardship programs for rational use of essential antimicrobials in animal farms to improve food safety and prevent the emergence of MDR bacteria.

## Data availability statement

The datasets presented in this study can be found in online repositories. The names of the repository/repositories and accession number(s) can be found in the article/Supplementary material.

## Author contributions

WZ, BT, ZY, and MY: conceptualization. HY, MY, and BT: funding acquisition. WZ, ZZ, HL, JWu, YD, HY, HJ, and BT: investigation. WZ, ZZ, and BT: methodology. ZY, BT, and MY: supervision. RL, JWa, XZ, and BT: visualization. RL and WZ: writing–original draft. All authors have read and agreed to the published version of the manuscript.

## Funding

This work was supported by the Key Research and Development Program of Zhejiang Province (2020C02031), the Key Research and Development Program of Hangzhou (202203A08), the earmarked fund for China Agriculture Research System (CARS-42-27), the State Key Laboratory for Managing Biotic and Chemical Threats to the Quality and Safety of Agro-Products (2010DS700124-ZZ2102), and Collaborative Extension Plan of Major Agricultural Technologies in Zhejiang Province (2021XTTGXM03).

## Conflict of interest

The authors declare that the research was conducted in the absence of any commercial or financial relationships that could be construed as a potential conflict of interest.

## Publisher’s note

All claims expressed in this article are solely those of the authors and do not necessarily represent those of their affiliated organizations, or those of the publisher, the editors and the reviewers. Any product that may be evaluated in this article, or claim that may be made by its manufacturer, is not guaranteed or endorsed by the publisher.

## Footnotes

^1^ https://github.com/tseemann/abricate

^2^ http://www.genomicepidemiology.org/services/

## References

[ref1] Bankevich A. Nurk S. Antipov D. Gurevich A. A. Dvorkin M. Kulikov A. S.. (2012). SPAdes: a new genome assembly algorithm and its applications to single-cell sequencing. J. Comput. Biol. 19, 455–477. doi: 10.1089/cmb.2012.0021, PMID: 22506599 PMC3342519

[ref2] Berne C. Ducret A. Hardy G. G. Brun Y. V. (2015). Adhesins involved in attachment to abiotic surfaces by gram-negative bacteria. Microbiol Spectr 3:10. doi: 10.1128/microbiolspec.MB-0018-2015, PMID: 26350310 PMC4566860

[ref3] Bojesen A. M. Ahmed U. Skaarup H. Espinosa-Gongora C. (2022). Recurring outbreaks by the same *Escherichia coli* ST10 clone in a broiler unit during 18 months. Vet. Res. 53:2. doi: 10.1186/s13567-021-01017-6, PMID: 35000591 PMC8744217

[ref4] Boolchandani M. D'Souza A. W. Dantas G. (2019). Sequencing-based methods and resources to study antimicrobial resistance. Nat. Rev. Genet. 20, 356–370. doi: 10.1038/s41576-019-0108-4, PMID: 30886350 PMC6525649

[ref5] Brisola M. C. Crecencio R. B. Bitner D. S. Frigo A. Rampazzo L. Stefani L. M.. (2019). *Escherichia coli* used as a biomarker of antimicrobial resistance in pig farms of southern Brazil. Sci. Total Environ. 647, 362–368. doi: 10.1016/j.scitotenv.2018.07.438, PMID: 30081373

[ref6] Chang J. Tang B. Chen Y. Xia X. Qian M. Yang H. (2020). Two IncHI2 plasmid-mediated Colistin-resistant *Escherichia coli* strains from the broiler chicken supply chain in Zhejiang Province, China. J. Food Prot. 83, 1402–1410. doi: 10.4315/jfp-20-041, PMID: 32294180

[ref7] Chen C. Chen H. Zhang Y. Thomas H. R. Frank M. H. He Y.. (2020). TBtools: an integrative toolkit developed for interactive analyses of big biological data. Mol. Plant 13, 1194–1202. doi: 10.1016/j.molp.2020.06.009, PMID: 32585190

[ref8] Deng H. Sun J. Ma J. Li L. Fang L. X. Zhang Q.. (2014). Identification of the multi-resistance gene cfr in *Escherichia coli* isolates of animal origin. PLoS One 9:e102378. doi: 10.1371/journal.pone.0102378, PMID: 25036029 PMC4103833

[ref9] Fu D. Wu J. Gu Y. Li Q. Shao Y. Feng H.. (2022). Corrigendum to the response regulator OmpR contributes to the pathogenicity of avian pathogenic *Escherichia coli*. Poult. Sci. 101:101876. doi: 10.1016/j.psj.2022.101876, PMID: 35393150 PMC9023887

[ref10] Gardner S. N. Slezak T. Hall B. G. (2015). kSNP3.0: SNP detection and phylogenetic analysis of genomes without genome alignment or reference genome. Bioinformatics 31, 2877–2878. doi: 10.1093/bioinformatics/btv271 25913206

[ref11] Gu H. Cai X. Zhang X. Luo J. Zhang X. Hu X.. (2021). A previously uncharacterized two-component signaling system in uropathogenic *Escherichia coli* coordinates protection against host-derived oxidative stress with activation of hemolysin-mediated host cell pyroptosis. PLoS Pathog. 17:e1010005. doi: 10.1371/journal.ppat.1010005, PMID: 34653218 PMC8550376

[ref12] Guan C. Tang B. Yang H. Ma J. Huang Y. Liu C. (2022). Emergence of plasmid-mediated tigecycline resistance gene, *tet*(X4), in *Escherichia fergusonii* from pigs. J Glob Antimicrob Resist 30, 249–251. doi: 10.1016/j.jgar.2022.06.029, PMID: 35793774

[ref13] He T. Wang R. Liu D. Walsh T. R. Zhang R. Lv Y.. (2019). Emergence of plasmid-mediated high-level tigecycline resistance genes in animals and humans. Nat. Microbiol. 4, 1450–1456. doi: 10.1038/s41564-019-0445-2, PMID: 31133751

[ref14] Hu Y. Yang X. Li J. Lv N. Liu F. Wu J.. (2016). The bacterial mobile resistome transfer network connecting the animal and human microbiomes. Appl. Environ. Microbiol. 82, 6672–6681. doi: 10.1128/aem.01802-16, PMID: 27613679 PMC5086561

[ref15] Li Y. Ed-Dra A. Tang B. Kang X. Müller A. Kehrenberg C.. (2022a). Higher tolerance of predominant *Salmonella* serovars circulating in the antibiotic-free feed farms to environmental stresses. J. Hazard. Mater. 438:129476. doi: 10.1016/j.jhazmat.2022.129476, PMID: 35809365

[ref16] Li Y. Kang X. Ed-Dra A. Zhou X. Jia C. Müller A.. (2022b). Genome-based assessment of antimicrobial resistance and virulence potential of isolates of non-pullorum/gallinarum *Salmonella* serovars recovered from dead poultry in China. Microbiol Spectr 10:e0096522. doi: 10.1128/spectrum.00965-22, PMID: 35727054 PMC9431532

[ref17] Li R. Xie M. Zhang J. Yang Z. Liu L. Liu X.. (2017). Genetic characterization of *mcr-1*-bearing plasmids to depict molecular mechanisms underlying dissemination of the colistin resistance determinant. J. Antimicrob. Chemother. 72, 393–401. doi: 10.1093/jac/dkw411, PMID: 28073961

[ref18] Lin J. Tang B. Zheng X. Chang J. Ma J. He Y.. (2022). Emergence of Incl2 plasmid-mediated colistin resistance in avian *Escherichia fergusonii*. FEMS Microbiol. Lett. 369:fnac016. doi: 10.1093/femsle/fnac016, PMID: 35157074

[ref19] Liu Y. Y. Wang Y. Walsh T. R. Yi L. X. Zhang R. Spencer J.. (2016). Emergence of plasmid-mediated colistin resistance mechanism MCR-1 in animals and human beings in China: a microbiological and molecular biological study. Lancet Infect. Dis. 16, 161–168. doi: 10.1016/s1473-3099(15)00424-7, PMID: 26603172

[ref20] Ma J. Zhou W. Wu J. Liu X. Lin J. Ji X.. (2022). Large-scale studies on antimicrobial resistance and molecular characterization of *Escherichia coli* from food animals in developed areas of eastern China. Microbiol Spectr 10:e0201522. doi: 10.1128/spectrum.02015-22, PMID: 35950758 PMC9430128

[ref21] Meng Q. Bai X. Zhao A. Lan R. Du H. Wang T.. (2014). Characterization of Shiga toxin-producing *Escherichia coli* isolated from healthy pigs in China. BMC Microbiol. 14:5. doi: 10.1186/1471-2180-14-5, PMID: 24393167 PMC3893481

[ref22] Paixão A. C. Ferreira A. C. Fontes M. Themudo P. Albuquerque T. Soares M. C.. (2016). Detection of virulence-associated genes in pathogenic and commensal avian *Escherichia coli* isolates. Poult. Sci. 95, 1646–1652. doi: 10.3382/ps/pew087, PMID: 26976911

[ref23] Peng X. Ed-Dra A. Yue M. (2022). Whole genome sequencing for the risk assessment of probiotic lactic acid bacteria. Crit. Rev. Food Sci. Nutr. 1–19. doi: 10.1080/10408398.2022.2087174, PMID: 35694810

[ref24] Peng Z. Hu Z. Li Z. Zhang X. Jia C. Li T.. (2022). Antimicrobial resistance and population genomics of multidrug-resistant *Escherichia coli* in pig farms in mainland China. Nat. Commun. 13:1116. doi: 10.1038/s41467-022-28750-6, PMID: 35236849 PMC8891348

[ref25] Rutuja D. Ragini M. Dhananjaya S. Kayzad N. Appasaheb G. Tannaz B. (2018). Antibiotic resistance characterization of Environmental *E. coli* isolated from river Mula-Mutha, Pune District, India. Int. J. Environ. Res. Public Health 15:1247. doi: 10.3390/ijerph15061247, PMID: 29895787 PMC6025386

[ref26] Shen Z. Wang Y. Shen Y. Shen J. Wu C. (2016). Early emergence of *mcr-1* in *Escherichia coli* from food-producing animals. Lancet Infect. Dis. 16:293. doi: 10.1016/s1473-3099(16)00061-x, PMID: 26973308

[ref27] Sullivan M. J. Petty N. K. Beatson S. A. (2011). Easyfig: a genome comparison visualizer. Bioinformatics 27, 1009–1010. doi: 10.1093/bioinformatics/btr039, PMID: 21278367 PMC3065679

[ref28] Sun J. Chen C. Cui C. Y. Zhang Y. Liu X. Cui Z. H.. (2019). Plasmid-encoded *tet*(X) genes that confer high-level tigecycline resistance in *Escherichia coli*. Nat. Microbiol. 4, 1457–1464. doi: 10.1038/s41564-019-0496-4, PMID: 31235960 PMC6707864

[ref29] Tang B. Chang J. Cao L. Luo Q. Xu H. Lyu W.. (2019). Characterization of an NDM-5 carbapenemase-producing *Escherichia coli* ST156 isolate from a poultry farm in Zhejiang, China. BMC Microbiol. 19:82. doi: 10.1186/s12866-019-1454-2, PMID: 31023222 PMC6482550

[ref30] Tang B. Chang J. Chen Y. Lin J. Xiao X. Xia X.. (2022a). *Escherichia fergusonii*, an underrated repository for antimicrobial resistance in food animals. Microbiol Spectr 10:e0161721. doi: 10.1128/spectrum.01617-21, PMID: 35138151 PMC8826826

[ref31] Tang B. Chang J. Luo Y. Jiang H. Liu C. Xiao X.. (2022b). Prevalence and characteristics of the *mcr-1* gene in retail meat samples in Zhejiang Province, China. J. Microbiol. 60, 610–619. doi: 10.1007/s12275-022-1597-y, PMID: 35362896

[ref32] Tang B. Chang J. Zhang L. Liu L. Xia X. Hassan B. H.. (2020a). Carriage of distinct *mcr-1*-harboring plasmids by unusual serotypes of *Salmonella*. Adv. Biosyst. 4:e1900219. doi: 10.1002/adbi.201900219, PMID: 32293141

[ref33] Tang B. Tang Y. Zhang L. Liu X. Chang J. Xia X.. (2020b). Emergence of *fexA* in mediating resistance to Florfenicols in *Campylobacter*. Antimicrob. Agents Chemother. 64:e00260-20. doi: 10.1128/AAC.00260-20, PMID: 32366706 PMC7317992

[ref34] Tang B. Wang Y. Luo Y. Zheng X. Qin X. Yang H.. (2021a). Coexistence of *optrA* and *fexA* in *Campylobacter*. mSphere 6:e00125-21. doi: 10.1128/mSphere.00125-21, PMID: 33980673 PMC8125047

[ref35] Tang B. Wang J. Zheng X. Chang J. Ma J. Wang J.. (2022c). Antimicrobial resistance surveillance of *Escherichia coli* from chickens in the Qinghai plateau of China. Front. Microbiol. 13:885132. doi: 10.3389/fmicb.2022.885132, PMID: 35935206 PMC9354467

[ref36] Tang B. Yang H. Jia X. Feng Y. (2021b). Coexistence and characterization of Tet(X5) and NDM-3 in the MDR-*Acinetobacter indicus* of duck origin. Microb. Pathog. 150:104697. doi: 10.1016/j.micpath.2020.104697, PMID: 33347964

[ref37] Tatusova T. DiCuccio M. Badretdin A. Chetvernin V. Nawrocki E. P. Zaslavsky L.. (2016). NCBI prokaryotic genome annotation pipeline. Nucleic Acids Res. 44, 6614–6624. doi: 10.1093/nar/gkw569, PMID: 27342282 PMC5001611

[ref38] Teng L. Liao S. Zhou X. Jia C. Feng M. Pan H.. (2022). Prevalence and genomic investigation of multidrug-resistant *Salmonella* isolates from companion animals in Hangzhou, China. Antibiotics (Basel) 11:625. doi: 10.3390/antibiotics11050625, PMID: 35625269 PMC9137667

[ref39] Verma V. Gupta S. Kumar P. Rawat A. Singh Dhanda R. Yadav M. (2019). Efficient production of endotoxin depleted bioactive α-hemolysin of uropathogenic *Escherichia coli*. Prep. Biochem. Biotechnol. 49, 616–622. doi: 10.1080/10826068.2019.1591993, PMID: 30929584

[ref40] Wang J. Tang B. Lin R. Zheng X. Ma J. Xiong X.. (2022). Emergence of *mcr-1-* and *bla*_NDM-5_-harbouring IncHI2 plasmids in *Escherichia coli* strains isolated from meat in Zhejiang, China. J. Glob. Antimicrob. Resist. 30, 103–106. doi: 10.1016/j.jgar.2022.06.002, PMID: 35697210

[ref41] Wang Y. Tian G. B. Zhang R. Shen Y. Tyrrell J. M. Huang X.. (2017). Prevalence, risk factors, outcomes, and molecular epidemiology of *mcr-1*-positive *Enterobacteriaceae* in patients and healthy adults from China: an epidemiological and clinical study. Lancet Infect. Dis. 17, 390–399. doi: 10.1016/s1473-3099(16)30527-8, PMID: 28139431

[ref42] Wangkheimayum J. Chanda D. D. Bhattacharjee A. (2022). Expression of *itaT* toxin gene is enhanced under aminoglycoside stress in *Escherichia coli* harbouring aac(6′)-Ib. Gene Reports 26:101526. doi: 10.1016/j.genrep.2022.101526

[ref43] Xu L. Wan F. Fu H. Tang B. Ruan Z. Xiao Y.. (2022). Emergence of Colistin resistance gene mcr-10 in *Enterobacterales* isolates recovered from fecal samples of chickens, slaughterhouse workers, and a nearby resident. Microbiol Spectr 10:e0041822. doi: 10.1128/spectrum.00418-22, PMID: 35412362 PMC9045214

[ref44] Yang L. Shen Y. Jiang J. Wang X. Shao D. Lam M.. (2022). Distinct increase in antimicrobial resistance genes among *Escherichia coli* during 50 years of antimicrobial use in livestock production in China. Nat. Food 3, 197–205. doi: 10.1038/s43016-022-00470-6 37117646

