# Corrigendum: Antimicrobial resistance and genomic characterization of *Escherichia coli* from pigs and chickens in Zhejiang, China

**DOI:** 10.3389/fmicb.2023.1102931

**Published:** 2023-02-10

**Authors:** Wei Zhou, Rumeng Lin, Zhijin Zhou, Jiangang Ma, Hui Lin, Xue Zheng, Jingge Wang, Jing Wu, Yuzhi Dong, Han Jiang, Hua Yang, Zhangnv Yang, Biao Tang, Min Yue

**Affiliations:** ^1^Zhejiang Provincial Center for Animal Disease Prevention and Control, Hangzhou, China; ^2^State Key Laboratory for Managing Biotic and Chemical Threats to the Quality and Safety of Agro-Products, Institute of Agro-Product Safety and Nutrition, Zhejiang Academy of Agricultural Sciences, Hangzhou, China; ^3^School of Food Science and Technology, Jiangnan University, Wuxi, China; ^4^Key Laboratory of Marine Food Quality and Hazard Controlling Technology of Zhejiang Province, China Jiliang University, Hangzhou, China; ^5^The Institute of Environment, Resource, Soil and Fertilizers, Zhejiang Academy of Agricultural Sciences, Hangzhou, China; ^6^Zhejiang Provincial Center for Disease Control and Prevention, Hangzhou, China; ^7^Department of Veterinary Medicine, Institute of Preventive Veterinary Sciences, Zhejiang University College of Animal Sciences, Hangzhou, Zhejiang, China

**Keywords:** *Escherichia coli*, animal origin, antimicrobial resistance, genomic characterization, virulence genes

A correction has been made to **Abstract**. This sentence previously stated:

“The AMR genes *bla*_NDM−5_ (1.10%, 2/181), *mcr-1* (1.10%, 2/181), *tet*(X4) (1.10%, 2/181), and *cfr* (6.08%, 2/181) were also found in these isolates.”

The corrected sentence appears below:

“The AMR genes *bla*_NDM−5_ (1.10%, 2/181), *mcr-1* (1.10%, 2/181), *tet*(X4) (1.10%, 2/181), and *cfr* (6.08%, 11/181) were also found in these isolates.”

The authors apologize for this error and state that this does not change the scientific conclusions of the article in any way. The original article has been updated.

