# Supplementary material for: Antimicrobial resistance and genomic characterization of Escherichia coli from pigs and chickens in Zhejiang, China
Source: Front Microbiol. 2022 Oct 24;13:1018682. doi: 10.3389/fmicb.2022.1018682 (PMC9638057; doi:10.3389/fmicb.2022.1018682)
Supplement: Supplementary file 1 [file Table_1.DOCX]

**Table S1.** The AMR patterns of 181 *E. coli* strains

| **ID** | **Antibiotic-resistant pattern** | **Number** | **Percentage (%)** |
| --- | --- | --- | --- |
| 1 | AMP-SPT-TET-FFC-SF-SXT | 31 | 17.13 |
| 2 | AMP-GEM-SPT-TET-FFC-SF-SXT-CEF-ENR-OFL | 14 | 7.73 |
| 3 | AMP-TET-FFC-SF-SXT | 13 | 7.18 |
| 4 | AMP-GEM-SPT-TET-FFC-SF-SXT-ENR-OFL | 10 | 5.52 |
| 5 | TET-SF-SXT | 7 | 3.87 |
| 6 | AMP-TET-FFC-SF-SXT-CEF-ENR-OFL | 6 | 3.31 |
| 7 | AMP-GEM-SPT-TET-FFC-SF-SXT-CEF | 6 | 3.31 |
| 8 | AMP-GEM-SPT-TET-FFC-SF-SXT | 5 | 2.76 |
| 9 | - | 4 | 2.21 |
| 10 | AMP--GEM-TET-FFC-SF-SXT | 4 | 2.21 |
| 11 | AMP-TET-SF-SXT | 4 | 2.21 |
| 12 | AMP-GEM--TET-FFC-SF-SXT-CEF--ENR-OFL | 4 | 2.21 |
| 13 | AMP-GEM-SPT-TET-FFC-SF-SXT-CEF-CAZ-ENR-OFL | 3 | 1.66 |
| 14 | AMP-SPT-TET-FFC-SF-SXT-CEF | 3 | 1.66 |
| 15 | AMP-SPT-TET-FFC-SF-SXT-CEF-ENR-OFL | 3 | 1.66 |
| 16 | AMP-SPT-TET-SF-SXT | 3 | 1.66 |
| 17 | AMP-GEM-TET-SF-SXT | 3 | 1.66 |
| 18 | SF | 3 | 1.66 |
| 19 | AMP-SPT-TET-FFC-SF-SXT-ENR-OFL | 2 | 1.10 |
| 20 | SPT-TET-FFC-SF-SXT | 2 | 1.10 |
| 21 | AMP-A/C-SPT-TET-FFC-SF-SXT | 2 | 1.10 |
| 22 | AMP-SPT-TET-FFC-SF-SXT-CEF-CAZ | 2 | 1.10 |
| 23 | AMP-A/C-GEM-SPT-TET-FFC-SF-SXT-CEF-CAZ-ENR-OFL | 2 | 1.10 |
| 24 | AMP-SPT-TET-FFC-SF-CEF-ENR-OFL | 2 | 1.10 |
| 25 | AMP-TET-FFC-SF-CEF-ENR-OFL | 2 | 1.10 |
| 26 | TET-FFC-SF | 2 | 1.10 |
| 27 | SF-SXT | 2 | 1.10 |
| 28 | TET-SF | 2 | 1.10 |
| 29 | AMP-GEM-SPT-TET-FFC-SF-SXT-CEF-OFL | 2 | 1.10 |
| 30 | FFC-SXT | 1 | 0.55 |
| 31 | SPT-TET-FFC-SF-SXT-ENR-OFL | 1 | 0.55 |
| 32 | SPT-TET-FFC-SF-SXT-ENR-OFL-CL | 1 | 0.55 |
| 33 | AMP-SPT-TET-FFC-SF-SXT-ENR | 1 | 0.55 |
| 34 | AMP-A/C-SPT-TET-FFC-SF-SXT-CEF-ENR-OFL | 1 | 0.55 |
| 35 | AMP-TET-FFC-SF-SXT-ENR-OFL | 1 | 0.55 |
| 36 | AMP-A/C--SPT-TET-FFC-SF-SXT-CEF-CAZ-ENR | 1 | 0.55 |
| 37 | AMP-GEM-SPT-TET-FFC-SF | 1 | 0.55 |
| 38 | AMP-FFC-SF-SXT-CEF | 1 | 0.55 |
| 39 | AMP-SPT-TET-FFC-SF-SXT-OFL | 1 | 0.55 |
| 40 | GEM-TET-SF-SXT | 1 | 0.55 |
| 41 | AMP-SPT-TET-FFC-SF-SXT-OFL-CL | 1 | 0.55 |
| 42 | SPT-TET-SF-SXT | 1 | 0.55 |
| 43 | AMP-SPT-TET-FFC-SF-SXT-ENR-CL | 1 | 0.55 |
| 44 | AMP-SPT-TET-FFC-SF-ENR-OFL | 1 | 0.55 |
| 45 | TET-FFC-SF-SXT | 1 | 0.55 |
| 46 | AMP-GEM--TET-FFC-SF-SXT-ENR-OFL | 1 | 0.55 |
| 47 | AMP-GEM-SPT-TET-FFC-SF-SXT-CEF-OFL | 1 | 0.55 |
| 48 | AMP-SPT-TET-SF-SXT-CEF | 1 | 0.55 |
| 49 | AMP-GEM-TET-FFC-SF-SXT-ENR-OFL-CL | 1 | 0.55 |
| 50 | AMP-GEM-SPT-CEF--ENR-OFL-CL | 1 | 0.55 |
| 51 | AMP-TET-FFC-SF--CEF--ENR-OFL-CL | 1 | 0.55 |
| 52 | AMP-SPT-TET-FFC-SF-SXT-CEF--ENR-OFL-CL | 1 | 0.55 |
| 53 | AMP-SF-CEF-CAZ-ENR-OFL | 1 | 0.55 |
| 54 | AMP-TET-FFC-SF--CEF-CAZ-ENR-OFL-MEM | 1 | 0.55 |
| 55 | AMP-GEM-SPT-FFC-SXT-CEF-CL | 1 | 0.55 |
| 56 | AMP-GEM-SPT-TET-FFC-SF-SXT-CEF-CAZ-ENR-OFL-CL | 1 | 0.55 |
| 57 | AMP | 1 | 0.55 |
| 58 | AMP-TET-FFC-SF | 1 | 0.55 |
| 59 | AMP-SF-SXT-CEF | 1 | 0.55 |
| 60 | AMP-TET-FFC-SF-SXT-CEF-CAZ-ENR-OFL | 1 | 0.55 |
| 61 | AMP-TET-FFC-SF-SXT-CEF-ENR-OFL-MEM | 1 | 0.55 |
| 62 | AMP-SF | 1 | 0.55 |
| 63 | AMP-SF-SXT | 1 | 0.55 |
| 64 | AMP-GEM-SPT-TET-FFC-SF-CEF-CAZ-ENR-OFL | 1 | 0.55 |

**Table S2.** The level of agreement between phenotypic-genotypic antimicrobial resistance

| **antibiotics** | **concordance rate (%)** | **A (%)** | **B (%)** | **C (%)** | **D (%)** |
| --- | --- | --- | --- | --- | --- |
| penicillins | 87.85 | 83.43 | 4.42 | 7.18 | 4.97 |
| aminoglycosides | 83.43 | 75.69 | 7.73 | 12.15 | 4.42 |
| cephalosporins | 87.29 | 0.00 | 87.29 | 0.00 | 12.71 |
| carbapenems | 100.00 | 1.10 | 98.90 | 0.00 | 0.00 |
| sulfonamides | 78.45 | 76.24 | 2.21 | 1.66 | 19.89 |
| quinolones | 58.01 | 45.30 | 12.71 | 17.13 | 24.86 |
| tetracyclines | 90.61 | 90.61 | 0.00 | 9.39 | 0.00 |
| chloramphenicols | 74.59 | 66.85 | 7.73 | 4.97 | 20.44 |
| polypeptides | 93.92 | 0.55 | 93.37 | 0.55 | 5.52 |

A: both genotype and phenotype were positive

B: both genotype and phenotype were negative

C: only genotypes of acquired AMR genes are present

D: only AMR phenotypes are present
